# Risk for Human African Trypanosomiasis, Central Africa, 2000–2009

**DOI:** 10.3201/eid1712.110921

**Published:** 2011-12

**Authors:** Pere P. Simarro, Giuliano Cecchi, José R. Franco, Massimo Paone, Eric M. Fèvre, Abdoulaye Diarra, José Antonio Ruiz Postigo, Raffaele C. Mattioli, Jean G. Jannin

**Affiliations:** World Health Organization, Geneva, Switzerland (P.P. Simarro, J.R. Franco, J.G. Jannin);; Food and Agriculture Organization, Rome, Italy (G. Cecchi, M. Paone, R.C. Mattioli);; University of Edinburgh, Edinburgh, UK (E.M. Fèvre);; World Health Organization, Brazzaville, Congo (A. Diarra);; World Health Organization, Cairo, Egypt (J.A. Ruiz Postigo)

**Keywords:** human African trypanosomiasis, Trypanosoma brucei gambiense, sleeping sickness, epidemiology, tropical medicine, medical geography, geographic information systems, parasites, central Africa

## Abstract

Comprehensive georeference records for human African trypanosomiasis in Cameroon, Central African Republic, Chad, Congo, Equatorial Guinea, and Gabon were combined with human population layers to estimate a kernel-smoothed relative risk function. Five risk categories were mapped, and ≈3.5 million persons were estimated to be at risk for this disease.

The most recent continental estimates of persons at risk for human African trypanosomiasis (HAT), also known as sleeping sickness, were published by the World Health Organization in 1998 (*1*). These estimates were provided on a country-by-country basis, and they were largely based on educated guesses and rough estimations of experts. Since that time, major progress has been made in HAT control and surveillance (*2*). Data collection and reporting have also substantially improved and increasingly include an explicit and accurate mapping component (*3**–**6*). The magnitude of the recent advances in HAT control and surveillance is such that up-to-date estimates of the number and distribution of persons at risk are urgently needed. The purpose of this study was to develop a method to estimate and map the risk for HAT in central Africa.

## The Study

The study area in central Africa comprised Cameroon, Central African Republic, Chad, Congo, Equatorial Guinea, and Gabon. The Gambian form of sleeping sickness caused by *Trypanosoma brucei gambiense* is endemic to these 6 countries.

Reported cases for 2000–2009 were obtained from the Atlas of HAT (*7*). This Atlas provided mapped data at the village level for 15,083 (94.2%) of 16,005 cases of HAT reported in the study region. Average mapping accuracy for these cases was ≈800 m (*7*). For the remaining 5.8% of cases, village-level information was unavailable, but the focus of geographic origin was known. Therefore, these cases were distributed among the disease-endemic villages of their focus by using proportional allocation, whereby proportionally more unmapped cases were attributed to mapped villages that reported more cases during the study period.

Human population distribution was obtained from LandScan databases (www.ornl.gov/sci/landscan). LandScan provides global grids in which census counts are allocated to grid nodes by probability coefficients (*8**,**9*). Probability coefficients were based on land cover, elevation, slope, roads, and populated areas/points. LandScan spatial resolution is <1 km at the equator, and population layers are updated yearly. To delineate risk areas, an average of all LandScan population layers during 2000–2009 was used. Subsequently, LandScan 2009 data were combined with the risk map to provide estimates of persons at risk at the end of the study period.

Both input layers (i.e., HAT cases and LandScan human population) can be regarded as spatial point processes, thus amenable to spatial smoothing (*10*). Smoothing results in intensity surfaces where the intensity of a point process is the mean number of events per unit area (*11*). For this study, the intensity of HAT cases and human population was estimated by using a quadratic kernel function (*12*). Intensity surfaces were generated by using the same search radius (*13*), which was set at 30 km.

Before spatial smoothing, the number of HAT cases reported in 2000–2009 was divided by 10 to yield the average number of cases per year. All LandScan layers from 2000 through 2009 were also averaged and subsequently converted from grid (raster format) to points (vector format) to enable spatial smoothing. No attempt was made to account for edge effects of smoothing (*11*), but edge effects were not expected to matter unduly because our final objective was to estimate the ratio of 2 intensities (*14*).

Spatial smoothing resulted in 2 surfaces, D(x,y) and P(x,y), which represented average annual estimates of disease intensity and population intensity, respectively. A relative risk function R(x,y) could subsequently be estimated as the ratio D(x,y)/P(x,y). Thresholds were applied to the function R(x,y) to distinguish 5 categories of risk ranging from very high to very low (Table 1). When R(x,y) was <1 HAT case/million persons/year, risk was considered marginal.

**Table 1:** Thresholds for definition of risk for human African trypanosomiasis, central Africa, 2000–2009

Category	No. cases/inhabitants/y
Very high	>1/10^2^
High	<1/10^2^ to >1/10^3^
Moderate	<1/10^3^ to >1/10^4^
Low	<1/10^4^ to >1/10^5^
Very low	<1/10^5^ to >1/10^6^

The results of the analysis are shown in the Figure, in which risk areas are mapped, and in Table 2, which summarizes the number of persons at risk, the extent of areas at risk, and the corresponding number of cases. In the 6 countries studied, ≈3.5 million persons are estimated to be at risk for contracting HAT (8.9% of the total population), distributed over an area of 224,000 km^2^ (7.5% of the total land area).

**Figure:**
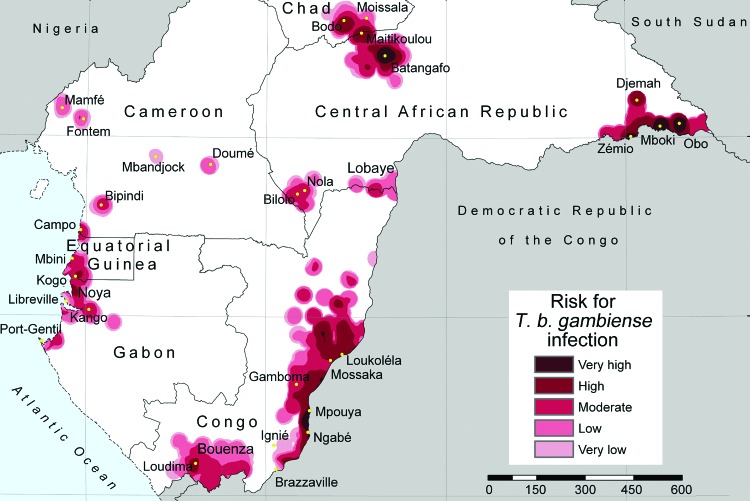
Lambert azimuthal equal-area projection (www.quadibloc.com/maps/maz0204.htm) of risk for infection with *Trypanosoma brucei gambiense*, central Africa, 2000–2009.

**Table 2:** Estimated risk for infection with *Trypanosoma brucei gambiense* in 6 countries, central Africa, 2000–2009*

Country	Risk category					Total
	Very high	High	Moderate	Low	Very low	
Cameroon	0 (0); 0	0 (0); 0	28 (22); 97	236 (80); 87	366 (71); 1	630 (172); 185
Central African Rep	29 (59); 6,075	33 (123); 1,193	115 (212); 287	133 (140); 28	65 (78); 0	374 (612); 7,583
Chad	0 (0); 0	109 (33); 2,884	110 (34); 76	120 (34); 19	119 (36); 1	458 (137); 2,980
Congo	3 (16); 1,250	105 (205); 2,266	479 (367); 1,169	443 (337); 68	138 (164); 3	1,168 (1,089); 4,756
Equatorial Guinea	0 (0); 0	2 (4); 42	27 (37); 130	8 (16); 1	4 (8); 0	40 (65); 173
Gabon	0 (0); 0	2 (6); 161	21 (58); 116	19 (69); 24	762 (36); 27	804 (168); 328
Total	32 (74); 7,325	251 (372); 6,546	780 (730); 1,875	959 (675); 227	1,453 (392); 32	3,475 (2,244); 16,005

*Values are no. persons x 10^3^ at risk (area in km^2^ × 10^2^ at risk); no. cases of human African trypanosomiasis. Risk categories are defined in Table 1. Rep, Republic.

Very high-risk areas comprise the most active foci in the Central African Republic (Batangafo, Obo, Mboki, and Zemio) and in Congo (Mpouya and Ngabé). These zones are located mainly in rural areas in which human population density is low, but they also include a few small towns. Areas included in the high-risk category are in foci in Bodo (Chad); Maitikoulou and Djemah (Central African Republic); Noya (Gabon); Kogo (Equatorial Guinea); and Loukoléla, Mossaka, Ignié, and Loudima (Congo). High-risk zones are also located around very high–risk areas. The moderate-risk category includes foci in Nola-Bilolo and Lobaye Prefecture (Central African Republic), Bipindi and Campo (Cameroon), Kango and Port Gentil (Gabon), Mbini (Equatorial Guinea), and areas in Bouenza and Gamboma (Congo). Low-risk areas were found mainly at the periphery of zones to which HAT is highly endemic, but they also include a few isolated foci with low levels of transmission, such as Mamfé, Fontem, and Doumé (Cameroon). Very low­–risk zones represent the extreme periphery of active foci, but they also include isolated rural foci, such as Mbandjock (Cameroon) and Libreville (Gabon), one of the largest urban agglomerations in the region.

## Conclusions

The methods reported provide an evidence-based approach to mapping the risk for HAT and estimating at-risk population. The use of different risk categories enables severity of the disease to be ranked.

We did not attempt to model underascertainment and underreporting, which are known to affect a neglected disease such as HAT. However, recent progress in the fields of active and passive surveillance (*3*) and comprehensive and systematic collection and mapping of HAT data over a 10-year period (*7*) substantially contributed to the robustness of the presented risk estimates. At the same time, we highlight that further improvements in consistency and coverage of HAT case detection and reporting are needed and require long-term efforts.

Because of the novel approach used in this study, it is unwarranted to make comparisons with previous figures of at-risk population, especially if the goal is to explore trends. Conversely, use of global human population layers and regular updating of the Atlas of HAT will enable future trends to be captured and the method to be applied to all countries to which HAT is endemic.

The type of risk maps presented will help target the most appropriate, site-specific strategies for HAT control and surveillance, such as the optimal frequency of active screening activities (*15*). These maps will enable limited resources available to be allocated rationally and where they are needed most.

## References

[1] World Health Organization. Control and surveillance of African trypanosomiasis. Geneva: The Organization; 1998. 10070249

[2] Simarro PP, Jannin J, Cattand P. Eliminating human African trypanosomiasis: where do we stand and what comes next? PLoS Med. 2008;5:e55. 10.1371/journal.pmed.0050055 18303943 PMC2253612

[3] Cecchi G, Paone M, Franco JR, Fèvre E, Diarra A, Ruiz J, Towards the Atlas of human African trypanosomiasis. Int J Health Geogr. 2009;8:15. 10.1186/1476-072X-8-15 19296837 PMC2661317

[4] Simarro PP, Diarra A, Ruiz Postigo JA, Franco JR, Jannin JG. The Human African Trypanosomiasis Control and Surveillance Programme of the World Health Organization 2000–2009: the way forward. PLoS Negl Trop Dis. 2011;5:e1007. 10.1371/journal.pntd.0001007 21364972 PMC3042999

[5] Cecchi G, Courtin F, Paone M, Diarra A, Franco JR, Mattioli RC, Mapping sleeping sickness in western Africa in a context of demographic transition and climate change. Parasite. 2009;16:99–106. 19585887 10.1051/parasite/2009162099

[6] Simarro PP, Franco JR, Cecchi G, Paone M, Diarra A, Ruiz JA. Human African trypanosomiasis in non-endemic countries (2000–2010). J Travel Med. 2011. In press. 10.1111/j.1708-8305.2011.00576.x 22221811

[7] Simarro PP, Cecchi G, Paone M, Franco JR, Diarra A, Ruiz JA, The atlas of human African trypanosomiasis: a contribution to global mapping of neglected tropical diseases. Int J Health Geogr. 2010;9:57. 10.1186/1476-072X-9-57 21040555 PMC2988709

[8] Bhaduri B, Bright E, Coleman P, Dobson J. LandScan: locating people is what matters. Geoinformatics. 2002;5:34–7.

[9] Dobson J, Bright E, Coleman P, Durfee R, Worley B. LandScan: a global population database for estimating populations at risk. Photogramm Eng Remote Sensing. 2000;66:849–57.

[10] Diggle PJ. Statistical analysis of spatial point patterns. London: Academic Press; 1983.

[11] Pfeiffer DU, Stevenson M, Robinson TP, Stevens KB, Rogers DJ, Clements AC. Spatial analysis in epidemiology. Oxford: Oxford University Press; 2008.

[12] Silverman BW. Density estimation for statistics and data analysis. New York: Chapman and Hall; 1986.

[13] Kelsall JE, Diggle PJ. Kernel estimation of relative risk. Bernoulli. 1995;1:3–16. 10.2307/3318678

[14] Bithell JF. An application of density estimation to geographical epidemiology. Stat Med. 1990;9:691–701. 10.1002/sim.4780090616 2218172

[15] Simarro PP, Franco JR, Ndongo P, Nguema E, Louis FJ, Jannin J. The elimination of *Trypanosoma brucei gambiense* sleeping sickness in the focus of Luba, Bioko Island, Equatorial Guinea. Trop Med Int Health. 2006;11:636–46. 10.1111/j.1365-3156.2006.01624.x 16640616

